# Radiation-Induced Gene Expression Changes in High and Low Grade Breast Cancer Cell Types

**DOI:** 10.3390/ijms19041084

**Published:** 2018-04-04

**Authors:** Valentina Bravatà, Claudia Cava, Luigi Minafra, Francesco Paolo Cammarata, Giorgio Russo, Maria Carla Gilardi, Isabella Castiglioni, Giusi Irma Forte

**Affiliations:** 1 Institute of Molecular Bioimaging and Physiology, National Research Council, 90015 Cefalù (Pa), Italy; valentina.bravata@ibfm.cnr.it (V.B.); luigi.minafra@ibfm.cnr.it (L.M.); francesco.cammarata@ibfm.cnr.it (F.P.C.); giorgio.russo@ibfm.cnr.it (G.R.); mariacarla.gilardi@ibfm.cnr.it (M.C.G.); giusi.forte@ibfm.cnr.it (G.I.F.); 2 Institute of Molecular Bioimaging and Physiology, National Research Council, 20090 Segrate (Mi), Italy; claudia.cava@ibfm.cnr.it

**Keywords:** ionizing radiation, breast cancer, gene expression profile, pathway analysis

## Abstract

Background: There is extensive scientific evidence that radiation therapy (RT) is a crucial treatment, either alone or in combination with other treatment modalities, for many types of cancer, including breast cancer (BC). BC is a heterogeneous disease at both clinical and molecular levels, presenting distinct subtypes linked to the hormone receptor (HR) status and associated with different clinical outcomes. The aim of this study was to assess the molecular changes induced by high doses of ionizing radiation (IR) on immortalized and primary BC cell lines grouped according to Human epidermal growth factor receptor (HER2), estrogen, and progesterone receptors, to study how HR status influences the radiation response. Our genomic approach using in vitro and ex-vivo models (e.g., primary cells) is a necessary first step for a translational study to describe the common driven radio-resistance features associated with HR status. This information will eventually allow clinicians to prescribe more personalized total doses or associated targeted therapies for specific tumor subtypes, thus enhancing cancer radio-sensitivity. Methods: Nontumorigenic (MCF10A) and BC (MCF7 and MDA-MB-231) immortalized cell lines, as well as healthy (HMEC) and BC (BCpc7 and BCpcEMT) primary cultures, were divided into low grade, high grade, and healthy groups according to their HR status. At 24 h post-treatment, the gene expression profiles induced by two doses of IR treatment with 9 and 23 Gy were analyzed by cDNA microarray technology to select and compare the differential gene and pathway expressions among the experimental groups. Results: We present a descriptive report of the substantial alterations in gene expression levels and pathways after IR treatment in both immortalized and primary cell cultures. Overall, the IR-induced gene expression profiles and pathways appear to be cell-line dependent. The data suggest that some specific gene and pathway signatures seem to be linked to HR status. Conclusions: Genomic biomarkers and gene-signatures of specific tumor subtypes, selected according to their HR status and molecular features, could facilitate personalized biological-driven RT treatment planning alone and in combination with targeted therapies.

## 1. Introduction

Breast cancer (BC) is the second most common cause of death from cancer among women worldwide [1]. In recent years, many treatment options for BC have been developed such as surgery, endocrine therapy, chemotherapy, and radiation therapy (RT), often chosen in combination, as recently described by several authors, e.g., [2,3].

Approximately 50% of all cancer patients receive RT (e.g., by external beams, intraoperative electron radiotherapy -IOERT, or internal RT), and extensive scientific evidences support the use of RT as a vital component of multimodal therapy for many types of cancer. In BC, RT is an efficient treatment for controlling localized tumors, often described as the last option for patients with inoperable cancers or the first choice in cases of incompletely resected or recurrent tumors after surgery [4]. In particular, IOERT is a therapeutic technique consisting of a single high dose of ionizing radiation (IR) administered immediately after surgical removal of a tumor to destroy residual cancer cells that may be left in the tumor site and that typically represent a high risk of cancer recurrence. According to specific eligibility criteria, IOERT may be delivered either as an anticipated boost of 9 Gy, followed by conventional external RT to guarantee optimal dose delivery, or as an exclusive single radiation dose of 23 Gy, corresponding to the administration of the entire sequence of conventional adjuvant RT [5,6].

BC is a heterogeneous disease at both clinical and molecular levels, presenting distinct subtypes (i.e., Luminal A, Luminal B, Triple Negative, HER2+) [7] associated with different clinical outcomes. Histologically, BC is classified by the immunohistochemistry assessment of hormone receptors (HR), specifically estrogen (ER) and progesterone (PR) receptors, and human epidermal growth factor receptor (HER2) [8].

Although technological advances in radiation delivery have strongly enhanced the tumor killing capacity, the current clinical scenario still offers standard RT protocols for groups of patients with the same cancer type (e.g., BC, prostate cancer), regardless of the molecular subtypes. However, it is well established that tumor heterogeneity, in terms of clinical and molecular characteristics, strongly affects the outcome of RT. Thus, delivering the same RT to all BC patients may not be the best option. Considering that biological differences in the radio-sensitivity of tumor subtypes are not taken into account when determining the total dose and the number of fractions administered during RT schedules, we argue that more radiobiological research is needed to understand and control the response variability. For example, the triple-negative BC subtype (ER−/PR−/HER2−) is described as a highly aggressive tumor with strong metastatic abilities and poor sensitivity to treatments, including RT [9].

RT treatment planning is currently based exclusively on radiation dose and tumor volume. However, in the post-genomic era, the possibility of including molecular information on the tumor to be treated could increase the therapeutic efficacy by enhancing the tumor control probability and better controlling the normal tissue complication probability. Molecular information would make it possible to prescribe a higher total dose for more radio-resistant tumors and a lower total dose for more sensitive tumors, with a consequent reduction of side effects [10].

To this end, research teams need to make great efforts to help clinicians to understand the molecular IR response of different cancer subtypes, so that the best RT modality and schedule, and the potential combination with other targeted treatment modalities, can be prescribed according to the specific molecular characterization of the tumor to be treated.

Specific gene signatures have been used to predict radio-sensitivity in many cancer types, including BC [11,12,13]. Several studies have reported the effects of hormones, cytokines, and antineoplastic drugs on ER and PR levels, but, to the best of our knowledge, limited data are available on the effect of IR on HR-related pathways [14,15,16,17,18,19,20,21,22].

The delivery of IR during RT schedules induces many pathways that control the survival/apoptosis balance by activating the key genes that regulate the cell cycle, survival, DNA repair, and inflammation [8,23]. Understanding the complex relationships among these processes may open up new avenues for radiation research and therapy to optimize and personalize RT treatment.

The aim of the present study was to assess the molecular differences, in particular at the gene expression and pathway level, induced by sub-lethal doses of IR treatment (9 Gy, corresponding to the anticipated boost IOERT treatment, followed by conventional external RT treatment) and by 23 Gy of IR in groups of BC cell lines with different HR statuses.

For this purpose, three human immortalized cell lines (MCF10A, MCF7, and MDA-MB-231) and three primary cell cultures (HMEC, BCpc7, and BCpcEMT), obtained from both tumor and healthy specimens, were classified into three groups of BC: a high grade group (with ER−/PR−/HER2−), a low grade group (with ER+/PR+/HER2−), and a group originating from healthy tissue. We assessed alterations in gene expression levels and enriched pathways after the two IR treatments in both immortalized and primary cell cultures.

Our findings highlight the involvement of specific gene and pathways signatures related to different HR statuses, which are probably linked to differences in IR cell responses. Our opinion is that such molecular signatures of specific tumor subtypes, selected according to histological and molecular features, could facilitate the planning of personalized biological-driven RT treatment, alone or in combination with targeted therapies, to increase cancer radio-sensitivity.

## 2. Results

### 2.1. Gene Expression and Pathway Analysis

For IR-treated (9 and 23 Gy) immortalized and primary cells, results of gene expression differential analysis are provided in Supplementary Figures S1 and S2. Table 1 reports the results of Venn diagram analysis comparing differentially expressed genes (DEGs) and enriched pathways in the different experimental conditions. The lists of genes and pathways are provided in Supplementary Table S1 and Supplementary Table S2, respectively.

#### 2.1.1. 9 Gy-Treated Immortalized Breast Cell Lines

Differential gene expression analysis of immortalized breast cell lines versus untreated cells revealed that irradiation significantly altered the expression levels of more than 400 genes selected in total by 2-fold or more (Table 1: 560 for MCF10A, 777 for MCF7, and 416 for MDA-MB-231, respectively). More details can be found in Supplementary Figures S1 and S2 and Supplementary Table S1.

For 9 Gy, Table 1 shows few DEGs in common with respect to the total DEGs between the immortalized breast cell lines (<10%). Thirty-two DEGs (<8%) were found in common between the two tumorigenic cell lines (MCF7 and MDA-MB-231). However, no pathway has been found in common after gene enrichment, suggesting a cell-line specific response to RT. Indeed, a much larger number of DEGs (from 357 to 685, from 86% to 88%) and enriched pathways (10–19) is observed in each breast cell line as a different effect of RT. Among these pathways we found, for MCF7 cell line, the p53 and DNA damage-inducible factor pathways, for MDA-MB-231 cell line, meiotic synapsis, and meiotic recombination, and pathways of chromosome and telomere maintenance.

Overall, these results suggest that the immortalized cell lines have a different response to IR, thus indicating that the action of the RT can be cell line-dependent.

#### 2.1.2. 23 Gy-Treated Immortalized Breast Cell Lines

Similarly to the results of 9 Gy-treated immortalized breast cell lines versus untreated cells, 23 Gy irradiation significantly altered the expression levels of more than 500 genes selected in total by 2-fold or more (Table 1: 582 for MCF10A, 505 for MCF7, and 627 for MDA-MB-231, respectively), (more details in Supplementary Figures S1 and S2 and Supplementary Materials Table S1).

As for the 9 Gy, even for 23 Gy, few DEGs (7–47, <8%) were found in common between the immortalized cell lines. Some pathways (12) were found between the tumorigenic cell lines. However, a cell line-specific response to RT is confirmed by the higher number of DEGs (from 415 to 548, from 82% to 88%) in each breast cell line as different effect of RT.

Overall, the results obtained for the 23 Gy-treated immortalized cell lines confirmed the results obtained from the 9 Gy-treated immortalized cell lines, showing a cell-line specific response to RT.

#### 2.1.3. 9 Gy-Treated Primary Breast Cell Cultures

Differential gene expression analysis revealed that more than 400 irradiated genes have expression levels significantly altered by 2-fold or more compared with the untreated reference group (Table 1, Supplementary Figures S1 and S2 and Supplementary Table S1).

Some DEGs were found in common between normal and tumorigenic cell cultures (23–73, 5–17%). We obtained 194 common DEGs (37%) between the two primary BC cell cultures (BCpc7 and BCpcEMT) and other 84 DEGs (20%) between the three breast primary cell cultures. Among these 84 DEGs we found Cell division cycle associated 5 (*CDCA5*), Cell division cycle 6 (*CDC6*), Cell division cycle associated 7 (*CDCA7*), E2F transcription factor 1 (*E2F1*), E2F transcription factor 2 (*E2F2*), Polo like kinase 4 (*PLK4*), Minichromosome maintenance 10 (*MCM10*), and Minichromosome maintenance 6 (*MCM6*).

Interestingly, 27 enriched pathways (including kinesins, repair, cell cycle checkpoints, and MCM pathways) were activated in both the two tumorigenic primary cultures (while no common pathway was found in immortalized breast cancer cell lines). Other 15 enriched pathways (including cell cycle, DNA replication, telomere maintenance, and DNA synthesis pathways) were found in common in all the three primary breast cell cultures.

A cell line-specific response to RT is shown also for the primary breast cell cultures by the high number of DEGs in each breast cell line (from 226 to 336, from 43% to 49%) as a different effect of RT. Specifically, among the 25 pathways found for ER/PR positive BC cell lines (BCpc7), we identified transcription and degradation of mitotic protein pathways. The apoptosis pathway was uniquely activated in the triple-negative BC primary cultures (BCpcEMT).

Overall, the primary breast cultures showed a more homogeneous response to IR, since RT appeared to activate a shared molecular response profile (common pathways), although the primary cells showed a distinct response to RT.

#### 2.1.4. 23 Gy-Treated Primary Breast Cell Cultures

Similarly to results already observed for 9 Gy, 23 Gy irradiation significantly altered the expression levels of hundreds of genes (more than six hundred) selected in total by 2-fold or more (Table 1, Supplementary Figures S1 and S2 and Supplementary Table S1).

Although many genes were deregulated only in specific breast primary cell cultures (249–370, 40–49%), we observed 101 common DEGs (16%) between the two primary BC cell cultures (BCpc7 and BCpcEMT) and other 203 (33%) in common between all three primary breast cell cultures. After enrichment, we identified 2 pathways (p53 and cell cycle pathways) that were activated only in BC primary cultures and other 33 pathways in common in all three primary breast cell cultures.

Overall, 23 Gy RT appears to activate a shared molecular response profile, although the primary cells show a distinct IR response.

### 2.2. Comparison between 9Gy and 23Gy

In order to assess the molecular response to the two different IR doses, for each cell line and primary breast cells, we compared the pathways enriched with DEGs after 9 Gy and 23 Gy irradiation (Table 1, last column).

Results show a good overlap in immortalized cell lines (MCF10A: 2/10 pathways; MCF7: 7/19 pathways; MDA-MB-231: 14/14 pathways) and a substantial overlap in breast primary cell cultures. (HMEC: 16/17 pathways; BCpc7: 58/68 pathways; BCpcEMT: 33/43 pathways).

### 2.3. Comparison between 9-Gy IR Treated Immortalized and Primary Cell Lines

Considering the good overlap of activated molecular pathways observed in response to 9 Gy and 23 Gy (see Results in Section 2.2), in this section we focused only on the molecular response of cells with similar HR status to 9 Gy IR, since this is a sub-lethal dose that is decisive for the cell fate decision (cell survival/ death balance).

In the nontumorigenic immortalized cell line (MCF10A) and healthy primary cell cultures (HMEC), only 11 DEGs were found in common (2,5%) (Table 1, last three rows).

More DEGs (64, 12%) were found in common in the low grade tumorigenic cell groups, e.g., *CDC6*, Cyclin dependent kinase inhibitor 1A (*CDKN1A*), Fanconi anemia complementation group A (*FANCA*), *MCM6*, Minichromosome maintenance (*MCM8*), *MCM10*, Fas cell surface death receptor (*FAS*) and MDM2 proto-oncogene (*MDM2*), while 59 DEGs (14%) were shared in the high grade tumorigenic groups (MDA-MB-231 and BCpcEMT).

After gene enrichment analysis, only 1 pathway (the peptide ligand binding receptor pathway) was found activated in the non-tumorigenic group samples; 6 pathways were shared between the low-grade tumorigenic group samples (Mitotic-G1-S phases, S-phase, cell cycle, activation of the pre-replicative complex, G1-S-transition, and p53 signaling pathway), and 9 pathways were in common in the high-grade group samples (Cell cycle, Chromosome maintenance, Deposition of new CENPA containing nucleosomes at the centromere, Telomere maintenance, Meiotic recombination, Meiosis, Meiotic synapsis, Packaging of telomere ends, and Amyloids).

#### PCA Analysis

Considering the gene signature consisting of (a) the 64 DEGs in common between MCF7 and BCpc7, and (b) the 59 DEGs in common between MDA-MB-231 and BCpcEMT, we performed a Principal Component Analysis.

**Figure 1:**
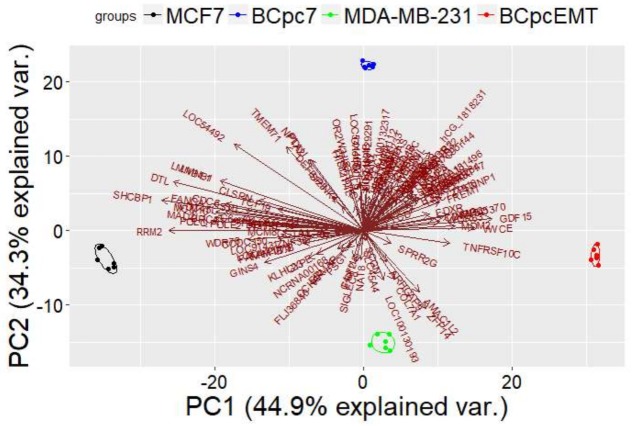
Principal component analysis considering gene signature shared in two breast cancer immortalized cell lines (MCF7 and MDA-MB-231) and in two BC primary cell cultures (BCpc7 and BCpcEMT).

Figure 1 presents the result of this analysis, showing that the gene signature was able to separate the different tumorigenic cells.

### 2.4. Network Analysis of 9 Gy DEGs in Immortalized and Primary Cells 

**Figure 2:**
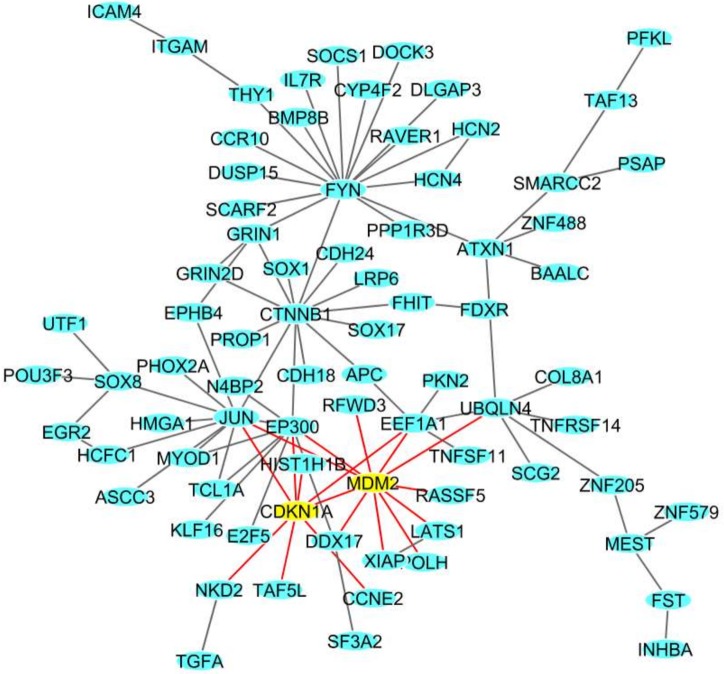
The deregulated densest protein-protein network (blue nodes: proteins, yellow nodes: proteins involved in crucial pathways, red edges: interactions involved yellow nodes) in the MCF10A breast cell line: nodes represent genes and edges represent protein interactions.

In MCF10 cells, among the total 560 DEGs (Table 1), we identified a network of 78 DEGs (Figure 2). Among these DEGs we found *CDKN1A*, involved in cell cycle progression and whose expression is controlled by p53, and *MDM2*, which is involved in the DNA damage pathway [24].

**Figure 3:**
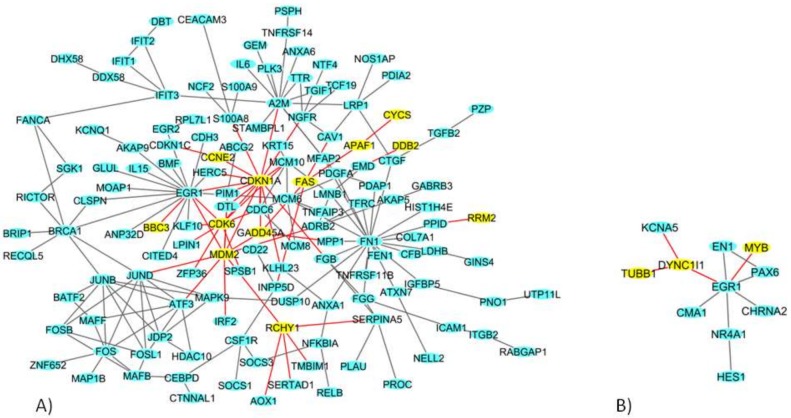
The deregulated densest protein-protein network (blue nodes: proteins, yellow nodes: proteins involved in crucial pathways, red edges: interactions involved yellow nodes) in (**A**) MCF7 BC and (**B**) MDA-MB-231 BC cell lines: nodes represent genes and edges represent protein interactions.

In MCF7 cells, among the total 777 DEGs (Table 1), we identified a network of 138 DEGs (Figure 3A). In particular, 12 genes in the network play a role in the p53 signaling pathway: Apoptotic peptidase activating factor 1 (*APAF1*), BCL2 binding component 3 (*BBC3*), Cyclin E2 (*CCNE2*), Cyclin dependent kinase 6 (*CDK6*), *CDKN1A*, Cytochrome c, somatic (*CYCS*), damage specific DNA binding protein 2 (*DDB2*), FAS, Growth arrest and DNA damage inducible factor (*GADD45S*), *MDM2*, Ribonucleotide reductase regulatory subunit M2 (*RRM2*), and Ring finger and CHY zinc finger domain-containing 1 (*RCHY1*) [24].

In MDA-MB-231 cells, among the total 416 DEGs (Table 1), we identified a network of 11 DEGs genes (Figure 3B), among which Tubulin beta 1 class VI (*TUBB1*), MYB proto-oncogene, transcription factor (*MYB*) and Dynein, cytoplasmic 1, and intermediate chain 1 (*DYNC1l1*) are involved in the cell cycle.

**Figure 4:**
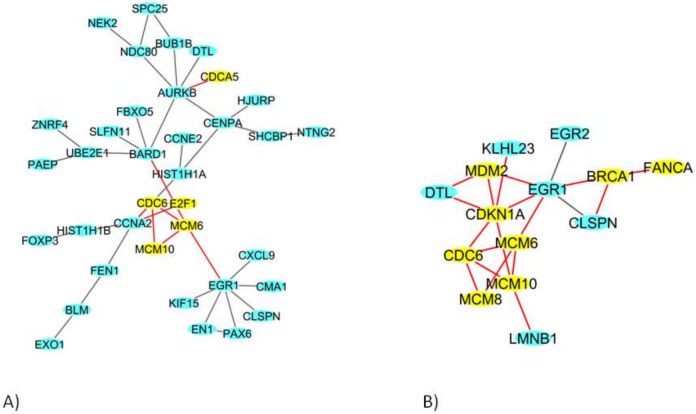
The deregulated densest protein-protein network (blue nodes: proteins, yellow nodes: proteins involved in crucial pathways, red edges: interactions involved yellow nodes) in (**A**) HMEC primary cells and (**B**) BCpc7 BC primary cells: nodes represent genes and edges represent protein interactions.

**Figure 5:**
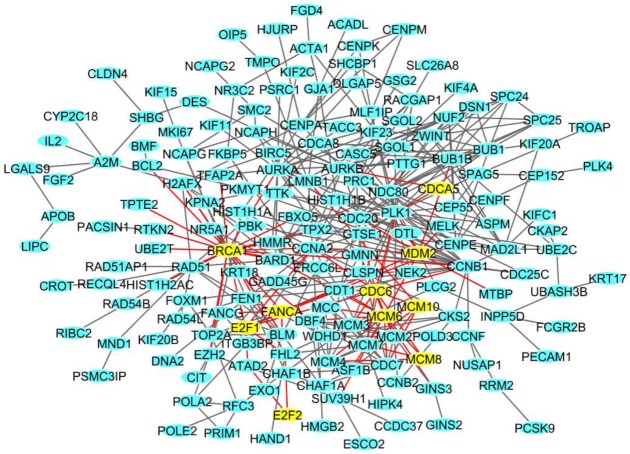
The deregulated densest protein-protein network (blue nodes: proteins, yellow nodes: proteins involved in crucial pathways, red edges: interactions involved yellow nodes) in BCpcEMT BC primary cells: nodes represent genes and edges represent protein interactions.

In HMEC healthy cells, among the 435 DEGs, we identified a network of 36 DEGs (Figure 4A); in BCpc7, among the 527 DEGs, a network of 214 DEGs was obtained (Figure 4B shows the genes more involved in the network), and a network of 175 DEGs was identified for BCpcEMT (Figure 5).

In these networks, we found genes involved in transcription (*E2F1*, *E2F2*), in the cell cycle (*CDCA5*, *CDC6*), in DNA replication (*MCM6*, *MCM8*, *MCM10*), in DNA repair (*BRCA1*, *FANCA*), and in DNA damage (*MDM2*, *CDKN1A*).

## 3. Discussion

RT is considered one of the current best strategies for the treatment of numerous malignant tumors, alone or in combination with other treatment modalities such as surgery, chemotherapy, and hormonal therapy.

Despite the technological advances made in recent decades, which permit the IR to be deposited with high precision on the tumor target, RT plans still prescribe the same total dose per organ tumor, without taking into account the biological differences attributable to the different tumor subtypes [10]. However, differences in the molecular portraits of specific cancer subtypes cause considerable variability in the response to radiation. Therefore, biological information on the tumor to be treated is necessary to select an appropriate treatment plan and thus achieve more successful treatment outcomes.

IOERT is a therapeutic technique in which a single high dose of IR is administered immediately after surgical removal of the tumor to destroy any cancer cells left in the tumor site. The purpose of this study was to investigate the radiation-induced gene expression profiles after IR doses of 9 and 23 Gy (doses relevant for IOERT) in nontumorigenic (MCF10A) and tumorigenic BC (MCF7 and MDA-MB-231) immortalized cell lines, and in healthy (HMEC) and BC (BCpc7 and BCpcEMT) primary cultures obtained from surgical biopsies. More precisely, as previously described, the samples were divided into three groups according to their HR status (low grade, high grade, and healthy groups), irradiated, and analyzed by cDNA microarray technology to select and compare their differential gene and pathway expressions. Although preliminary results of partial breast irradiation with IOERT, either as an anticipated boost (9 Gy) or as an exclusive treatment (23 Gy), seem to be promising in terms of local disease control, there is little information on the biological basis of the effects of IOERT. In the context of this radiation treatment modality, this study aimed to describe the molecular response, in terms of deregulated genes and pathways, to high doses of IR according to the different BC subtypes. Our genomic approach using in vitro and ex-vivo models (e.g., primary cells) is a necessary first step for a translational study to describe the common radio-resistance features associated with HR status. Such information would support clinicians in prescribing more personalized total doses or associated targeted therapies for specific tumor subtypes, thus enhancing cancer radio-sensitivity. However, this type of investigation would need to be extended to numerous panels of cell lines; thus, the present work should be considered as a pilot study.

Here, we report substantial alterations in gene expression levels after RT in both immortalized and primary cell cultures.

Considering the good overlap of activated molecular pathways observed in response to 9 and 23 Gy (Section 2.2), we focused on the 9 Gy treatment to compare the DEG and pathway lists of IR-treated immortalized and primary cell lines and to perform network analysis, as 9 Gy is a sub-lethal dose that is decisive for the cell fate decision (cell survival/death balance).

Irradiation with 9 Gy caused a change in the expression level of more than 700 genes in the MCF7 BC cell line (belonging to the low grade group) and about 400 genes in the MDA-MB-231 BC cells (high grade group) (Table 1). These results are congruent with published findings [25].

As previously described by our group and several other authors, radiation effects on cells are heterogeneous and appear to act in a cell line-dependent way. This behavior was also confirmed by the pathway analysis conducted in this study. More precisely, no common pathway was shared among the considered immortalized cell lines after IR 9 Gy (Table 1). Instead, IR activated specific molecular processes in a cell-line dependent way. Specifically, in the MCF7 cell line, the 9 Gy IR-related DNA damage response was highlighted, driven by the p53 pathway (*APAF1*, *BBC3*, *CCNE2*, *CDK6*, *CDKN1A*, *CYCS*, *DDB2*, *FAS*, *GADD45S*, *MDM2*, *RRM2*, and *RCHY1* found in the MCF7 network have a role in this pathway), specifically by the damage-specific DNA-binding protein (e.g., *DDB2*) and by DNA damage-inducible factors (e.g., *GADD45A*).

These results are in line with others reported in the literature [26] and also with data previously obtained by our group in a gene expression profile comparison study made of MCF7 cells exposed to a 9 Gy dose using electron and proton beams. The p53 signaling was found to be a driven pathway in the response to both types of radiation beams types, although it was probably activated by different genes (data submitted) [27]. The p53 pathway is often described as “the genome guardian,” because of its crucial role in the cell fate decision following IR-induced DNA damage. In particular, an interesting number of genes belonging to p53 signaling were deregulated in MCF7 cells after both electron and proton irradiation, some of which are involved in cell cycle arrest, DNA repair, and cell death processes. These genes are often described as deregulated after IR exposure and could be further investigated as a target of therapeutic combinatorial BC interventions. Moreover, in MDA-MB-231 cells, pathways involved in meiotic processes (e.g., meiotic synapsis and meiotic recombination) (*TUBB1*, *MYB*, and *DYNC1l1* found in the MDA-MB-231 network have a role in these pathways) and DNA maintenance processes (e.g., chromosome and telomere maintenance) were specifically deregulated after radiation exposure.

All of the primary cell cultures investigated here shared the activation of 15 common pathways (Table 1) that are often described in the literature as modulated in response to radiation. These pathways are involved, for instance, in the cell cycle, DNA replication, DNA repair, and DNA damage. They include the 84 DEGs encoding for cell division cycle proteins (*CDC6*, *CDCA5*, and *CDCA7*), transcription factors (*E2F1* and *E2F2*), *PLK4*, *MCM10*, and *MCM6*, and other cell cycle-associated proteins. Twenty-seven pathways are shared between the two tumorigenic primary cell cultures known to be activated in RT (e.g., DNA repair, cell cycle). However, a cell line-dependent RT response, clearly observed in this work in immortalized cell lines, has been found also in primary cell cultures, since 25 pathways including transcription and degradation of mitotic proteins were uniquely activated in BCpc7 and the apoptosis pathway in BCpcEMT.

Finally, we evaluated the DEGs lists and relative pathways enriched in immortalized and breast primary cultures according to HR status in the low grade, high grade, and healthy groups. As reported in Section 2.3, some IR-modulated genes and pathways were shared between samples belonging to the same HR-status group. More precisely, few genes (11) and only the peptide ligand binding receptor pathway were deregulated in the healthy samples.

In contrast, a signature of 64 DEGs and 6 common pathways were found to be deregulated in the low grade group samples, characterized by the positive status of ER and PR receptors: Mitotic-G1-S phases, S-phase, cell cycle, activation of the pre-replicative complex, G1-S-transition, and p53 signaling pathway. Overall, these cells are able to modulate processes involved in cell cycle regulation, repairing DNA strand breaks, and cell survival/death balance through the activation of apoptosis signaling. Interestingly, these results are congruent with others reported by our group, which highlighted the deregulation of genes controlling the cell cycle process [11,12,13,27].

A signature of 59 DEGs and 9 pathways were activated in the triple-negative cell lines of the high grade group samples. These pathways are involved in cell cycle regulation, nucleosomes, chromosome, and telomere maintenance, which could justify the more aggressive phenotypes and high rate of radio-resistance, because they are linked to chromosome instability, which is, in turn, caused by radiation exposure [28]. Interestingly, there is increasing attention being paid to the literature on direct targeted interventions against some key regulators of chromosome maintenance in BC samples. More precisely, some inhibitors of the key regulator of spindle formation, such as kinesin family member 1C (KIF1C), have been proposed as novel promising therapeutic strategies for the triple-negative BC subtype (in which the expression of this gene is known to be high) [29]. In addition, the activation of pathways related to telomere maintenance might contribute to the activation of specific cell processes, such as Stress Induced Premature Senescence. The induction of the cell senescence phenotype represents a permanent exit from the cell cycle; however, it is not a passive phenomenon, but instead consists of metabolic changes in protein expression and secretion that could induce transformation processes in cells predisposed to proliferation [30,31]. Indeed, as well described, senescence is able to induce radio-resistance by cell cycle regulation, alteration of apoptosis, and changes in the ability to repair DNA damage.

To the best of our knowledge, limited data are reported in the literature regarding the IR-induced gene expression changes in relation to BC tumor grading, especially in primary cultures. Although this is a pilot study, the identification of IR-induced low grade- and high grade-specific signaling may provide a source of biomarkers that is predictive of RT outcomes or that could be useful for the optimization of targeted therapies in tandem with RT [32,33,34,35,36].

## 4. Materials and Methods

### 4.1. Cell Cultures and Irradiation

Both immortalized and primary BC cell cultures were used. Immortalized cell lines, in particular, adenocarcinoma MCF7 and MDA-MB-231, which are characterized by different tumorigenic aggressive phenotypes [7] and non-tumorigenic mammary epithelial MCF10A cells, were purchased from the American Type Culture Collection and cultured according to the manufacturer’s instructions (ATCC, Manassas, VA, USA). Their genotypic differences and molecular information have been described in the literature [37,38]. Primary cell cultures were isolated from breast tissues of two patients with infiltrating ductal carcinoma (IDC), as previously described by our groups [39]. Briefly, the tumor tissue was mechanically and enzymatically disaggregated immediately after surgery, and primary cells were isolated by the differential centrifugation method. The BC primary cells were cultured in selective media and grown in standard conditions. The patients gave their written informed consent for research participation according to the Helsinki Declaration. The study was approved by the Ethical Committee of the San Raffaele G. Giglio Hospital, Cefalù (protocol number C.E. 2012/16; approval date 6 May 2009). The two BC primary cells were characterized by different phenotypes, hereafter named BCpc7 (ER+/PR+/HER2−) and BCpcEMT (ER−/PR−/HER2−) cells. We used human mammary epithelial cells (HMEC) as a healthy primary cell culture model, cultured according to the manufacturer’s instructions (Invitrogen, Carlsbad, CA, USA). Immortalized and primary cells seeded in 100-mm Petri dishes were maintained in an exponentially growing culture condition at 37 °C in a 5% CO_2_ incubator and were irradiated at sub-confluence under sterile conditions.

Cell irradiation treatments at different tissue depths were performed using the NOVAC7 (Sortina Iort Technologies, Vicenza, Italy) system. The electron accelerator system was calibrated under reference conditions. Cell radiation treatments were carried out at the doses of 9 and 23 Gy to the 100% isodose at a dose rate of 3.2 cGy/pulse. The cell irradiation setup and the dose distribution were conducted as previously reported [12].

We grouped both immortalized and primary cell lines into low grade and high grade groups, according to their histological features. More precisely, the low grade group contained immortalized MCF7 and primary BCpc7 BC cells (both ER+/PR+/HER2−), whereas the high grade group was formed by immortalized MDA-MB-231 and primary BCpcEMT BC cells (both ER−/PR−/HER2−) because of their more aggressive phenotype, as also previously described by our group [39,40,41]. Finally, immortalized MCF10A cells and primary HMEC were used as reference samples considering their healthy tissue origin. The immortalized and primary cell lines were tested for their radio-resistance to doses of 9 and 23 Gy, providing evidence for the major survival capacity of the HG group to high doses of IR [11,12,13,28].

### 4.2. Gene Expression Analysis

Gene expression profiling of immortalized cell lines (MCF10A, MCF7, and MDA-MB-231) and primary cell cultures (HMEC, BCpc7, and BCpcEMT) treated with electron beams using 9 and 23 Gy of IR were performed. Because IR exposure can cause time-dependent transcriptional changes, based on our previous experiences and several other studies this topic [12,13,42,43], we decided to analyze the gene expression profiles 24 h after the RT. The deregulation of gene expression trends and the activation of several cellular processes (such as DNA repair or chromatin organization) were extensively measured. Therefore, 24 h after each treatment, cells were harvested and counted, and the pellets were stored immediately at −80 °C.

Total RNA extraction and its qualitative and quantitative analyses were conducted as previously described [12,13]. Microarray analysis was performed using the Whole Human Genome 4 × 44 K cDNA kit with the Two-Color Microarray-Based approach according to the suggested protocol (Agilent Technologies, Santa Clara, CA, USA).

In particular, the experiment was performed using six replicates for each configuration assayed. Statistical data analysis, background correction, normalization, and summary of expression measures were conducted with one-sample *t*-tests for each breast cell line and breast cell culture, using R package (limma [44,45]) and an FDR-adjusted *p*-value of <0.01. Genes were identified as being differentially expressed genes (DEGs) if they showed a fold-change (FC) of at least 2 compared with untreated cells used as reference samples.

The data discussed in this publication have been deposited in the National Center for Biotechnology Information Gene Expression Omnibus (GEO) [46] and are accessible through GEO Series accession number (GSE108962). Microarray data are available in compliance with Minimum Information about a Microarray Experiment (MIAME) standards.

### 4.3. Pathway Analysis

Given that 1077 pathways were derived from the REACTOME [47], BIOCARTA [48], and KEGG [49] databases, a pathway enrichment analysis was applied. The enrichment was evaluated using Fisher’s Exact Test between the DEGs and all 1077 pathways [50]. All *p*-values were adjusted using the Benjamini-Hochberg procedure for multiple testing [50,51], and we considered a pathway to be enriched with DEGs if the adjusted *p*-value was <0.01. To display the results obtained for each DEG and enriched pathway, and all of the possible combinations between different breast cells, we constructed Venn diagrams [52]. We performed a Principal Component analysis using ggbiplot in R-package [53].

### 4.4. Network Analysis

The analysis of IR-deregulated biological networks was performed by creating protein-protein interactions for each cell line and primary culture. We downloaded protein-protein interactions from GeneMania using SpidermiR tool and identified the densest networks for each cell line and primary culture according to the networks with the highest numbers of connected DEGs find in total [54,55,56,57]. For each network, the nodes of the network represent the proteins, while the edges represent the interactions among them.

## 5. Conclusions

In this pilot study, we produced a comprehensive description of the effects of 9 and 23 Gy IR doses in BC and healthy immortalized and primary cell cultures. Overall, a great amount of reported data suggest that the response to radiation is cell-line dependent and that some specific gene and pathway signatures seem to be linked to HR status.

## Figures and Tables

**Table 1:** Results of Venn diagram analysis comparing differentially expressed genes (DEGs) and enriched pathways (PATHs) in the different experimental conditions: human immortalized cell lines (MCF10A, MCF7, and MDA-MB-231) and three primary cell cultures (HMEC, BCpc7, and BCpcEMT) after irradiation with 9 or 23 Gy.

	9 Gy		23 Gy		9 ∩ 23 Gy
	N° DEGs	N° PATHs	N° DEGs	N° PATHs	N° PATHs
**TOTAL MCF10A**	560	10	582	5	2
**TOTAL MCF7**	777	19	505	37	7
**TOTAL MDA-MB-231**	416	14	627	14	14
**MCF10A ∩ MCF7**	53	0	36	3	
**MCF10A ∩ MDA-MB-231**	20	0	25	0	
**MCF7 ∩ MDA-MB-231**	32	0	47	12	
**MCF10A ∩ MCF7 ∩ MDA-MB-231**	7	0	7	0	
**MCF10A-specific**	480	10	514	2	
**MCF7-specific**	685	19	415	22	
**MDA-MB-231-specific**	357	14	548	2	
**TOTAL HMEC**	435	17	658	54	16
**TOTAL BCpc7**	527	68	621	62	58
**TOTAL BCpcEMT**	687	43	753	35	33
**HMEC ∩ BCpc7**	23	1	68	21	
**HMEC ∩ BCpcEMT**	73	0	79	0	
**BCpc7 ∩ BCpcEMT**	194	27	101	2	
**HMEC ∩ BCpc7 ∩ BCpcEMT**	84	15	203	33	
**HMEC-specific**	255	1	308	0	
**BCpc7-specific**	226	25	249	6	
**BCpcEMT-specific**	336	1	370	0	
**MCF10A ∩ HMEC**	11	1			
**MCF7 ∩ BCpc7**	64	6			
**MDA-MB-231 ∩ BCpcEMT**	59	9			

## Supplementary Materials

Supplementary materials can be found at http://www.mdpi.com/1422-0067/19/4/1084/s1.

## Abbreviations

RT	Radiation therapy
BC	Breast Cancer
HR	Hormone receptor
HER2	Human epidermal growth factor receptor
IR	Ionizing radiation
ER	Estrogen receptor
PR	Progesteron receptor
IOERT	Intraoperative electron radiotherapy
DEG	Differentially expressed gene
